# Argon plasma coagulation for successful treatment of bile leakage after subtotal cholecystectomy

**DOI:** 10.1186/s40792-020-00876-z

**Published:** 2020-05-24

**Authors:** Tsukasa Aritake, Kenji Takagi, Natsuki Nagano, Ryutaro Kobayashi, Takashi Maeda, Kiyotaka Kawai, Satoru Kawai, Satoaki Kamiya

**Affiliations:** Department of Surgery, Tsushima City Hospital, 3-73, Tachibana Town, Tsushima City, Aichi 496-8537 Japan

**Keywords:** Argon plasma coagulation, Bile leakage, Subtotal cholecystectomy

## Abstract

**Background:**

Subtotal cholecystectomy is an effective surgical method to decrease the risk of complications for gallbladders that are difficult to remove. However, there is a risk for postoperative refractory bile leakage through the gallbladder stump. Here, we report a new management technique involving the use of argon plasma coagulation (APC) to stop bile leakage after a subtotal cholecystectomy.

**Case presentation:**

A 74-year-old man was referred to our hospital for abdominal pain and fever. Contrast-enhanced computed tomography of the abdomen showed fluid collection, such as an abscess, surrounding the gallbladder and hepatic flexure colon. The patient was diagnosed with colonic perforative peritonitis, and he underwent emergency surgery. On laparotomy, the abscess was observed outside of the hepatic flexure colon and gallbladder necrosis was detected. The neck of the gallbladder and the area close to the hepatoduodenal ligament was severely inflamed prohibiting dissection. The hepatic flexure colon was part of the abscess wall, and resection was needed. A subtotal cholecystectomy and right hemicolectomy confirmed peritonitis caused by cholecystic perforation. The mucous membrane of the gallbladder neck that remained was necrotic or detached. Therefore, the stump of the gallbladder was closed by primary sutures without cauterization of the mucosa. On postoperative day 6, bile leakage from the gallbladder stump was revealed. Percutaneous and endoscopic retrograde cholangiography drainage were performed. However, the liquid, which seemed to be secreted from the mucosa of the remnant gallbladder, was continuously obtained. We used APC to cauterize the gallbladder mucosa through the fistula of the abdominal drainage tube. Bile leakage and mucus discharge were improved after three rounds of APC cauterization.

**Conclusions:**

APC effectively treated refractory bile leakage from a gallbladder stump after subtotal cholecystectomy for severe cholecystitis.

## Background

Gallstone disease is a major health problem and often needs surgery. In the Western world, about 10–15% of adults will develop gallstones, with between 1 and 4% a year developing symptoms [1]. A “difficult gallbladder” is usually associated with severe inflammation that distorts the local anatomy and renders dissections more difficult (i.e., acute cholecystitis, empyema, gangrene, perforation, and Mirizzi syndrome) [2]. Subtotal cholecystectomy (SC) involves removal of the body and sometimes part of the infundibulum of the gallbladder, leaving the cystic duct not directly closed. SC may be performed when the structures of the Calot triangle cannot be identified and the Critical View of Safety method cannot be performed [2, 3]. SC effectively decreases the risks of complications, such as bile duct injury, hepatic artery injury, and bleeding from the gallbladder bed [3]. However, SC can lead to greater rates of postoperative bile leakage through the gallbladder stump, prolonged drainage, and an increased need for percutaneous or endoscopic retrograde cholangiographic drainage [3]. Up until now, there has been no defined management for difficult to cure cases. Here, we describe a new management technique we developed that cauterizes the gallbladder mucosa using argon plasma coagulation to treat bile leakage from the gallbladder stump after subtotal cholecystectomy.

## Case presentation

**Fig. 1:**
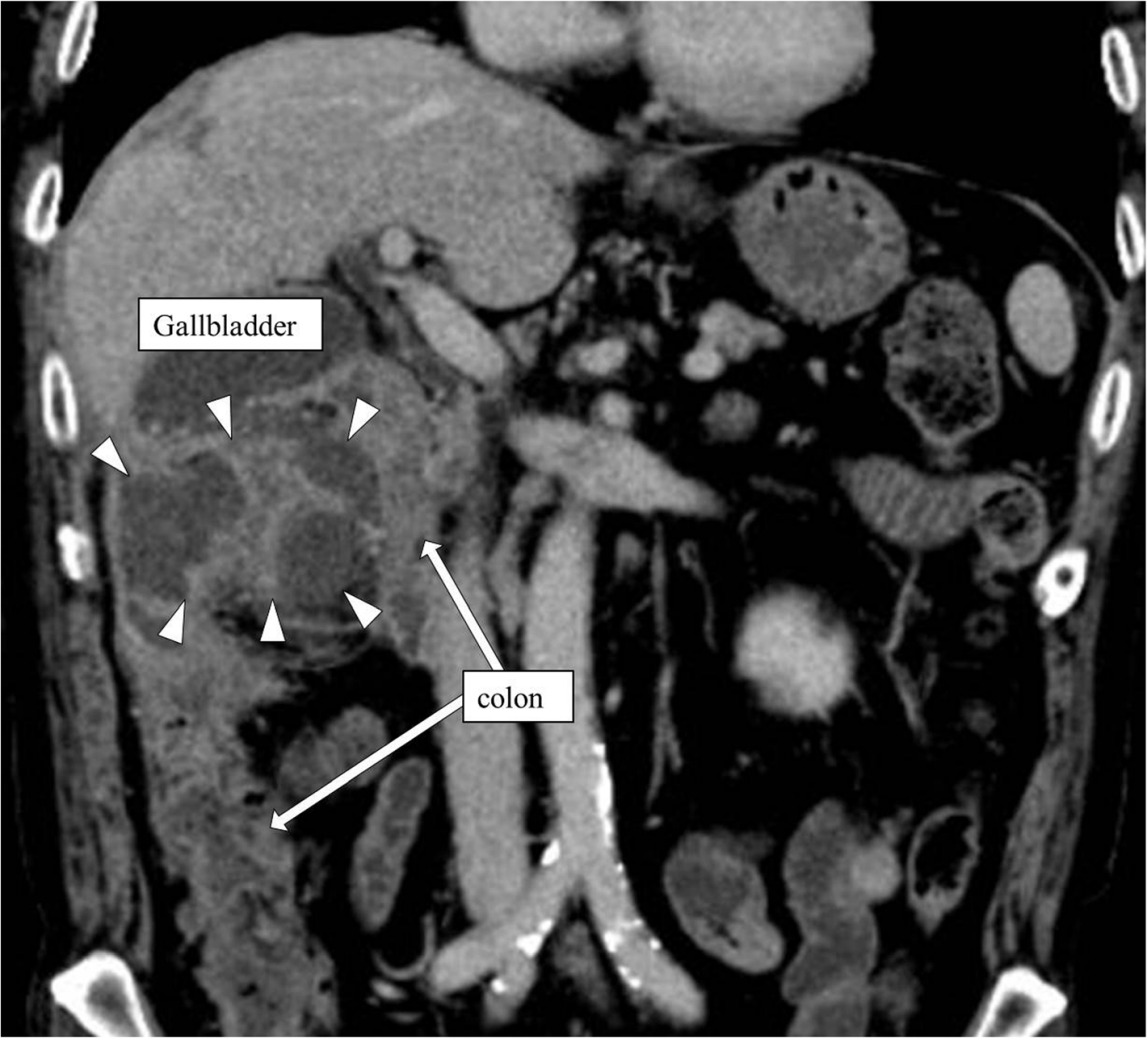
Contrast-enhanced CT of the abdomen showed fluid collection like abscess (white arrowhead) surrounded by gallbladder and hepatic flexure colon

A 74-year-old man was referred to our hospital for abdominal pain and fever. He had hypertension. He underwent surgery for rectal cancer 7 years ago and again for liver metastasis 2 years ago. He had a recurrence of liver metastasis and peritoneal dissemination a year ago and received chemotherapy (XELOX + bevacizumab). His blood tests showed an elevated white blood cell count of 15,300/mm^3^. A contrast-enhanced computed tomography (CT) of the abdomen showed fluid collection like an abscess surrounding the gallbladder and hepatic flexure colon (Fig. 1). The patient was diagnosed with colonic perforative peritonitis, and he underwent emergency surgery.

On laparotomy, the abscess was located outside of the hepatic flexure colon and necrosis was found on the gallbladder after mobilization of the ascending colon. The neck of the gallbladder and near the hepatoduodenal ligament was too severely inflamed to dissect. The hepatic flexure colon was part of the abscess wall, and resection was needed. For diagnosis of peritonitis caused by cholecystic perforation, a subtotal cholecystectomy and right hemicolectomy was performed. The gallbladder was removed at the neck, and the mucosa of the gallbladder neck was necrotic or detached. Therefore, the stump of the gallbladder was closed by primary sutures without cauterizing the mucosa. An abdominal drainage tube was placed into the gallbladder bed. The total operative time was 318 min, and the estimated blood loss was 1369 ml.

**Fig. 2:**
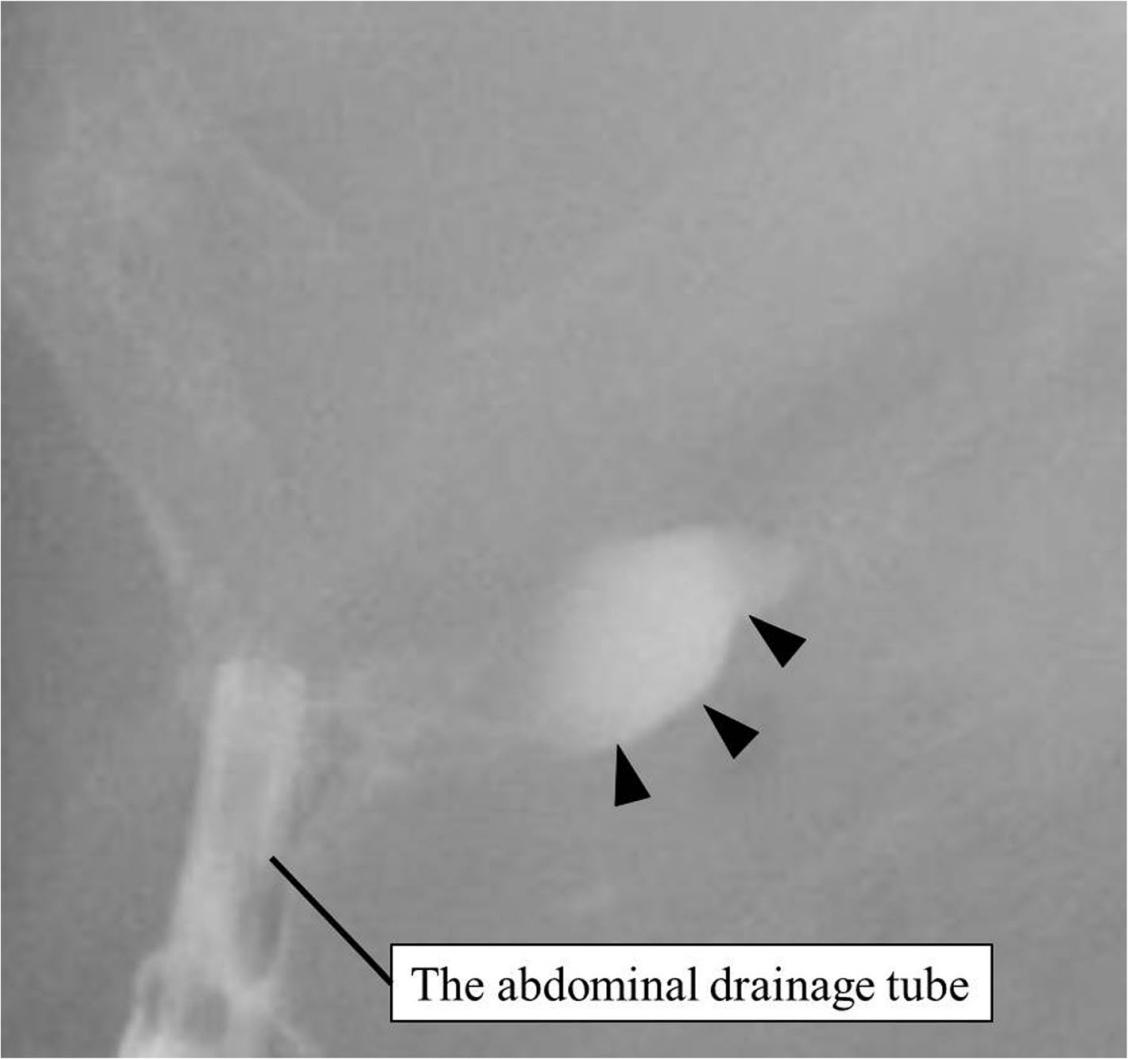
Contrast examination from the drainage tube enabled visualization of the neck of the gallbladder (black arrowhead)

**Fig. 3:**
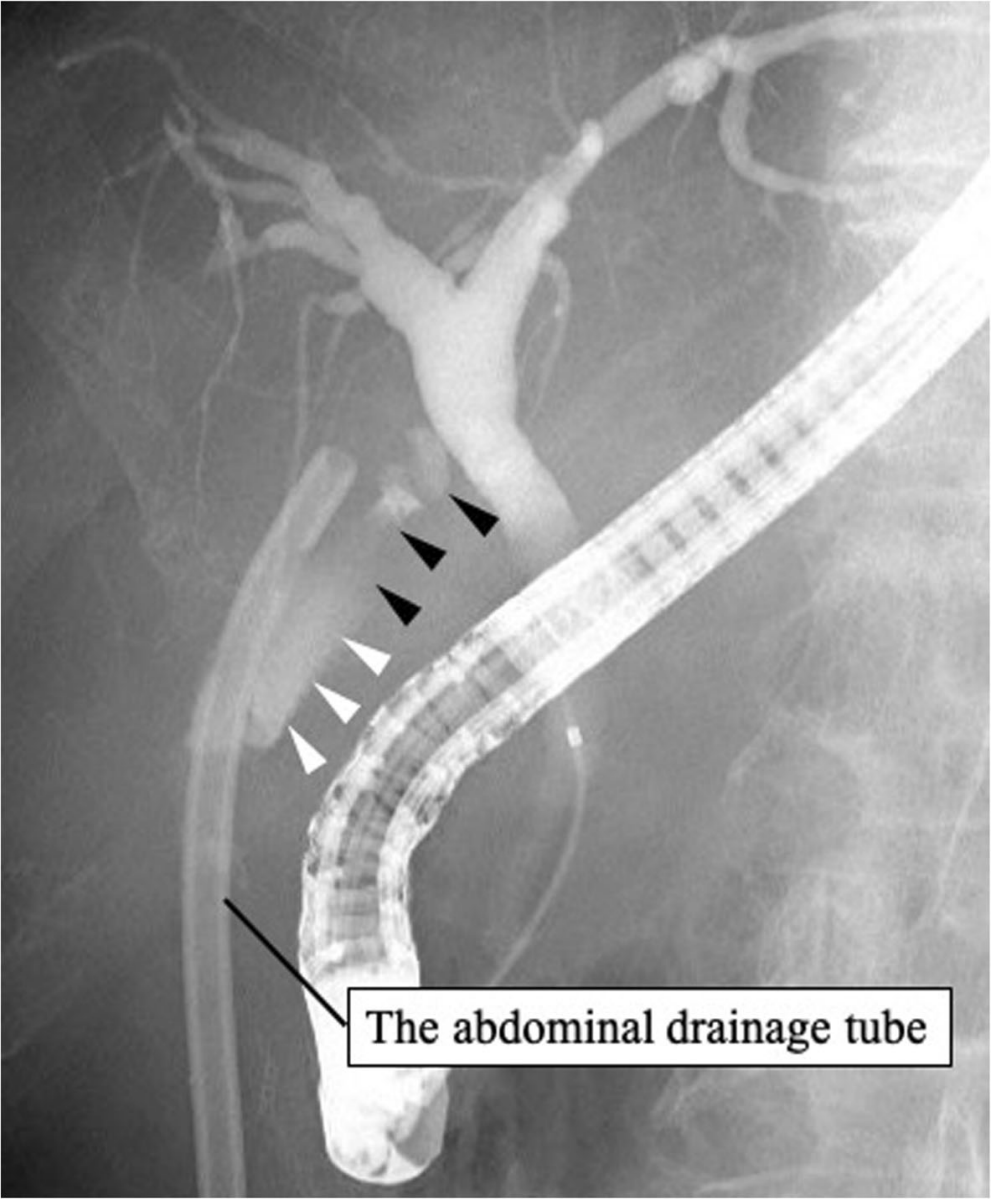
Imaging from ENBD tube showed the leakage (white arrowhead) from the gallbladder stump (black arrowhead)

On postoperative day (POD) 6, bile was detected from the abdominal drainage tube. Contrast examination of the drainage tube enabled visualization of the neck of the gallbladder, and bile leakage from the gallbladder stump was revealed (Fig. 2). On POD 29, bile leakage was still a problem and an endoscopic nasobiliary drainage (ENBD) tube was inserted. Imaging from the ENBD tube revealed the leakage was from the gallbladder stump (Fig. 3). After insertion of the ENBD tube, the volume from the abdominal drainage tube decreased and became less biliary, but a white transparent liquid was continuously obtained 100 ml or more per day. On POD 57, imaging from the ENBD tube enabled visualization of the remnant gallbladder and it revealed that the gallbladder stump and common bile duct were connected. The liquid seemed to be secreted from the mucosa of the remnant gallbladder. We planned to use argon plasma coagulation (APC) to cauterize the gallbladder mucosa through the fistula of the abdominal drainage tube. The patient was given a full explanation of the procedure, and written informed consent was obtained. All procedures used in this case report were approved by the ethical committee of our hospital.

**Fig. 4:**
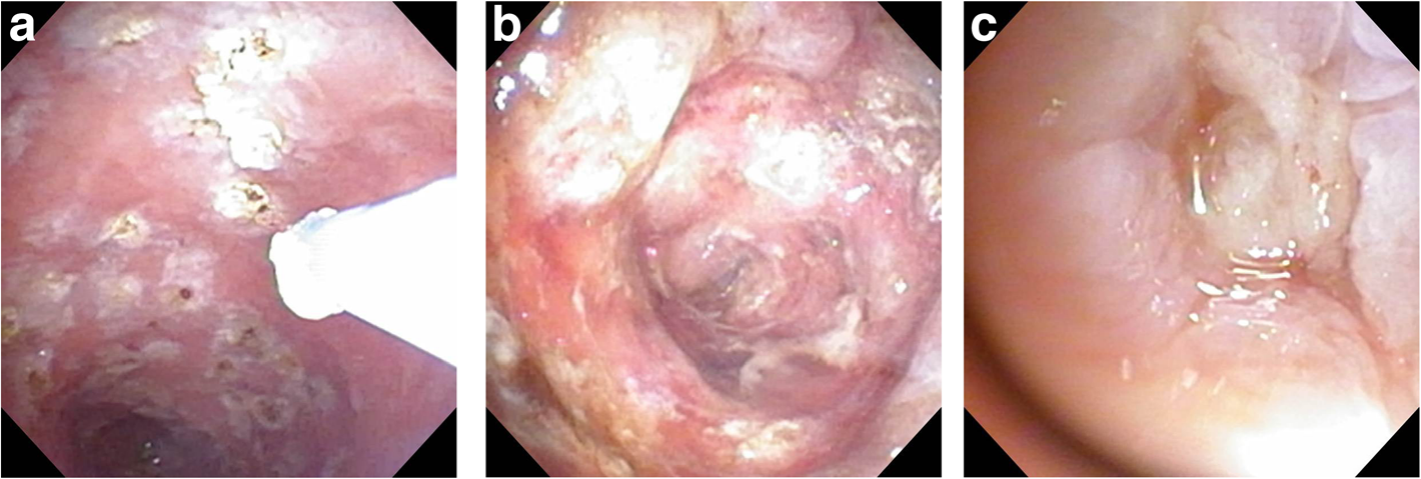
**a**–**c** Cholangioscopy findings. **a** At the first time, the membrane was cauterized several points randomly. **b** At the second time, the region cauterized at the first time was found sclerosis, and the other region was cauterized all over. **c** At the third time, a very small region of the membrane was found and cauterized

**Fig. 5:**
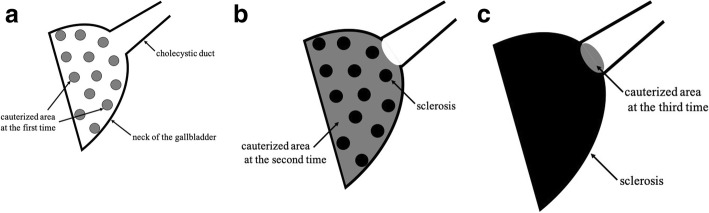
**a**–**c** Schemas of the gallbladder stump while APC cauterizing. **a** At the first time, the membrane was cauterized several points randomly. **b** At the second time, the region cauterized at the first time was found sclerosis, and the other region was cauterized all over. **c** At the third time, a very small region of the membrane was found and cauterized

**Fig. 6:**
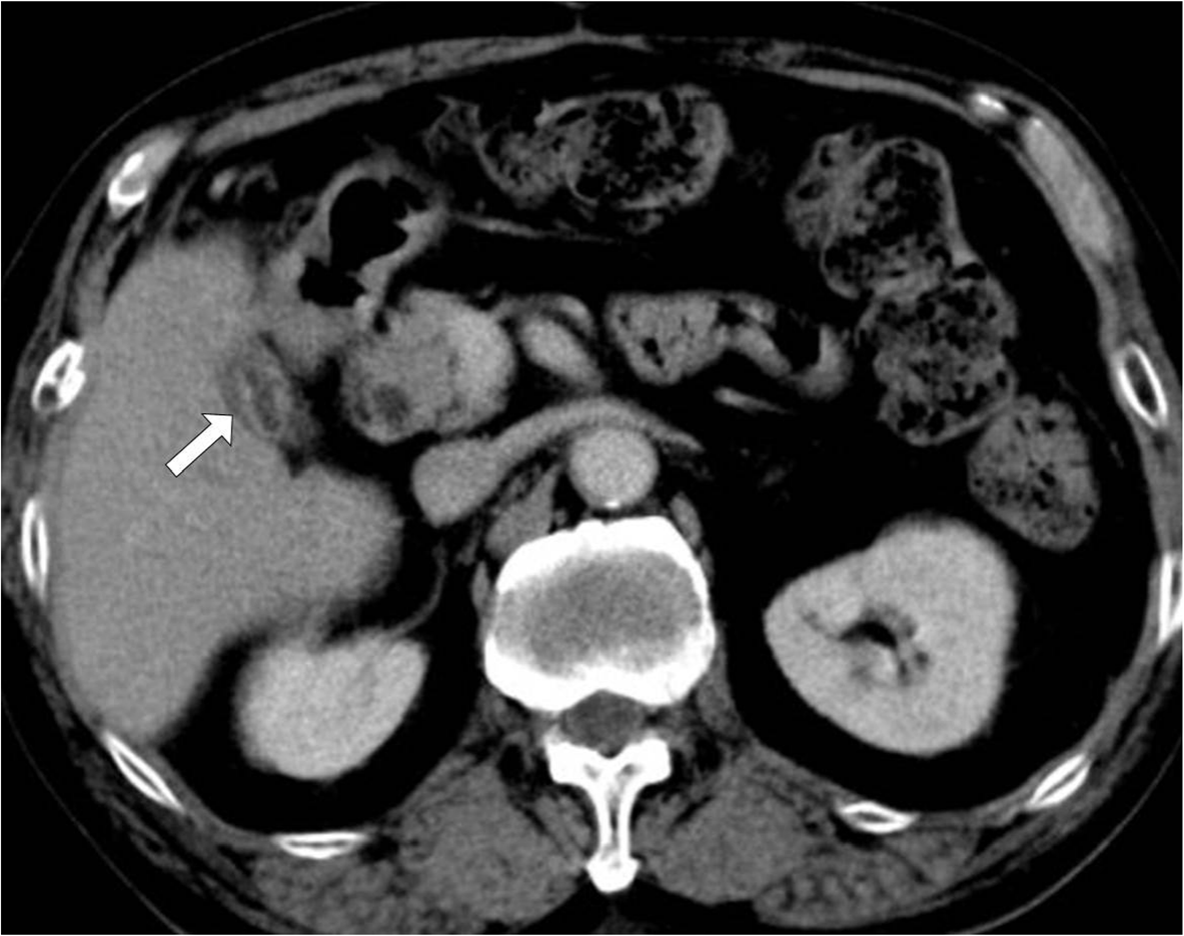
The follow-up CT scan 7 months after the surgery showed the left gallbladder was changed atrophic (white arrow)

APC cauterization was performed three times on PODs 64, 71, and 82 (Figs. 4 and 5). It was a video-assisted procedure using a CHF-U cholangioscope (Olympus Co., Tokyo, Japan), and the diameter of the scope was 5.2 mm. APC was performed with high frequency generator (VIO 300D), an automatically regulated argon source (APC2), and flexible APC probes (all manufactured by ERBE Elektromedizin, Tuebingen, Germany). We used argon gas at a flow rate of 1.5–2 L/min and a high-frequency arc output of 50–60 W. Cholangioscopy showed that the membrane of the remnant gallbladder was widely recognized, and entrance to the cholecystic duct was found in the back. Because the safety of APC cauterization of the gallbladder mucosa had not previously been reported, we initially tried to cauterize the membrane not entirely but randomly at several points. Upon the second cauterization, we found sclerosis in the region that was previously cauterized. There were no complications, and the second region was cauterized all over. A very small region of the membrane was cauterized on POD 82. The abdominal drainage volume decreased over time after removal of the ENBD tube on POD 87 and the abdominal drainage tube on POD 90. He was discharged on POD 95, and 7 months after the surgery, a follow-up CT scan showed the remnant gallbladder was atrophic (Fig. 6).

## Discussion

Madding first reported subtotal cholecystectomy in 1955 as an alternative for conventional total cholecystectomy as a rescue procedure in cases that were technically difficult to minimize the potential for injury to a severely inflamed bile duct and vascular structures [3, 4]. A subtotal cholecystectomy is an effective strategy to treat patients with a “difficult gallbladder” who have inflammation, fibrosis, and adhesions which increase the risk of complications with dissection of the cystic duct [5]. However, a recent systematic review showed that regardless of whether the gallbladder stump is closed, postoperative bile leakage occurs more frequently after a subtotal cholecystectomy than after a total cholecystectomy [2].

For treatment of bile leakage from the gallbladder stump, percutaneous drainage, endoscopic biliary drainage, and reoperation are often performed. When these treatments are not effective, another treatment is needed. Ethanol ablation, which requires injection of absolute ethanol into the causative isolated bile duct, has been used as a treatment for refractory bile leakage [6]. In our case, percutaneous or endoscopic biliary drainage was not effective. We considered ethanol ablation but felt it would be too difficult because the gallbladder stump was still connected to the common bile duct.

Therefore, we chose APC to cauterize the gallbladder mucosa. APC is a new method for coagulating tissue which employs a high-frequency electric current and ionized argon gas [7]. APC was first introduced during open surgery in the late 1970s, was adapted for use in endoscopy in 1991, and has become the most commonly used endoscopic coagulation technique [8]. APC treatment is characterized by non-contact coagulation, providing tangential irradiation to coagulate a target site uniformly [7]. APC was used for our case because we wanted to cauterize only the gallbladder mucosa and avoid damaging the deeper tissue, such as the common bile duct and vessels. Nakamura et al. reported that they used APC for treatment of gastric antral vascular ectasia without any complications [9], so we selected the same power setting as them. In our case, cauterizing the gallbladder by APC led to improvement of the mucus discharge and caused atrophy of the gallbladder stump. Furthermore, it is possibly implied that APC also improved bile leakage because of no recurrence of bile leakage after removing ENBD tube. Though most of the mucosa of the remnant gallbladder was cauterized by APC, the mucosa seemed to be somewhat enhanced in a follow-up CT scan. Even if the mucosa of the gallbladder regenerates a little, we concluded that the refractory mucus discharge was successfully treated by APC cauterizing.

With regard to complications, Sagawa et al. reported that only a sense of abdominal fullness was experienced by some patients who were treated early gastric cancer using APC, but was improved soon after completion of the treatment [7]. On the other hand, Wang et al. reported that some patients had severe complications involving rectal fistulation after treatment for hemorrhagic chronic radiation proctitis, and the only factor significantly associated with severe complications was ulcerated area greater than 1 cm^2^ [10]. They also recommended that rectums with ulcers developing after pelvic radiation could be considered as having fragile, ischemic, and poor healing tissue, which may partly explain why patients with large ulceration have a high risk of developing fistulation [10]. Therefore, APC treatment seems to be safe for early gastric cancer but less safe for hemorrhagic chronic radiation proctitis. In our case, no complications were experienced. Though there are few reports about APC treatment for bile leakage, it is suggested that APC is less invasive, safe, and an effective treatment method for refractory bile leakage from a gallbladder stump after subtotal cholecystectomy.

## Conclusion

APC was an effective treatment for refractory bile leakage from a gallbladder stump after subtotal cholecystectomy for severe cholecystitis in that APC allows for targeted cauterization, preventing damage to the surrounding tissues. However, it is necessary to carefully select cases because some complications have been known in APC treatment for other organs.

## Data Availability

Data sharing is not applicable to this article as no datasets were generated or analyzed during the present study.

## Abbreviations

APC: Argon plasma coagulation
CT: Computed tomography
POD: Post-operation day
SC: Subtotal cholecystectomy
ENBD: Endoscopic nasobiliary drainage
